# Targeting STAT3/miR-21 axis inhibits epithelial-mesenchymal transition via regulating CDK5 in head and neck squamous cell carcinoma

**DOI:** 10.1186/s12943-015-0487-x

**Published:** 2015-12-21

**Authors:** Shan-Shan Sun, Xuan Zhou, Yuan-Yuan Huang, Ling-Ping Kong, Mei Mei, Wen-Yu Guo, Ming-Hui Zhao, Yu Ren, Qiang Shen, Lun Zhang

**Affiliations:** The Maxillary Facial and Otorhinolaryngology Head & Neck Surgery, Tianjin Medical University Cancer Institute & Hospital, National Clinical Research Center for Cancer, Tianjin Key Laboratory of Cancer Prevention and Therapy, Huanhuxi Road, Tiyuanbei, Hexi District, Tianjin, TJ 300060 China; Basic Medical Research Center, Tianjin Medical University, Qixiangtai Road, Heping District, Tianjin, TJ 300070 China; Department of Clinical Cancer Prevention, Division of Cancer Prevention and Population Sciences, The University of Texas MD Anderson Cancer Center, Houston, TX 77030 USA

**Keywords:** CDK5, miR-21, EMT, HNSCC, Lymph node metastasis

## Abstract

**Background:**

Abnormal activation of STAT3 and miR-21 plays a vital role in progression and invasion of solid tumors. The cyclin-dependent kinase 5 (CDK5) is reported to contribute to cancer metastasis by regulating epithelial-mesenchymal transition (EMT). However, the role of STAT3/miR-21 axis and CDK5 in head and neck squamous cell carcinoma remains unclear.

**Methods:**

We measured the expression of miR-21, CDK5 and EMT markers in 60 HNSCC tumor samples. We used Immunohistochemistry and in situ hybridization assay to examine the role of STAT3/miR-21 axis and CDK5 activation in the invasiveness of HNSCC. The clinical survival relevance was analyzed by Kaplan-Meier analysis and univariate/multivariate COX regression model. Multiple approaches including scratch, transwell chamber assay and other molecular biology techniques were used to validate the anti-invasion effect of targeting miR-21 in Tca8113 and Hep-2 cell lines in vitro. Furthermore, whether miR-21 depletion inhibits HNSCC invasion in vivo was confirmed in Tca8113 xenograft tumor model.

**Results:**

The expression of miR-21 and CDK5 were significantly correlated with lymph node metastasis in HNSCC. Hep-2 and Tca8113 cell lines showed co-overexpression of miR-21 and CDK5. WP1066 or asON-miR-21 treatment depleted miR-21 and CDK5 expression and significantly inhibited migration or invasion in Hep-2 and Tca8113 cells. The expression levels of CDK5/p35, N-cadherin, vimentin, β-catenin were inhibited while E-cadherin level was increased by miR-21 depletion in vitro and in vivo*.* Conversely, ectopic CDK5 overexpression significantly induced tumor cell motility and EMT. Moreover, ectopic CDK5 overexpression in Hep-2 and Tca8113 cells rescued the observed phenotype after miR-21 silencing or WP1066 treatment.

**Conclusions:**

miR-21 cooperates with CDK5 to promote EMT and invasion in HNSCC. This finding suggests that CDK5 may be an important cofactor for targeting when designing metastasis-blocking therapy by targeting STAT3/miR-21 axis with STAT3 inhibitor or miR-21 antisense oligonucleotide. This is the first demonstration of the novel role of STAT3/miR-21 axis and CDK5/CDK5R1 (p35) in metastasis of HNSCC.

**Electronic supplementary material:**

The online version of this article (doi:10.1186/s12943-015-0487-x) contains supplementary material, which is available to authorized users.

## Background

Presently there are 600,000 new HNSCC patients worldwide annually [1]. Approximately up to 50 % HNSCC patients achieved 5-year overall survival, suggesting a poor mean survival for this cancer type [2]. Lymph node metastasis or other distant metastasis is one of the most significant factors responsible for the observed poor clinical outcome. Epithelial mesenchymal transition (EMT) is considered as a vital process of metastasis, and thus it may significantly contribute to the progression of HNSCC [3–5].

The oncogenic miR-21 gene is located in chromosome17q23.2 and is evolutionally conserved in vertebrates including humans. MiR-21 is known to play an essential role in regulating biological behaviors in many malignancies [6–8]. MiR-21 is also associated with several biomarkers and therapeutic targets in multiple epithelium cancers such as oral squamous cell carcinoma (OSCC) [9], renal cancer [10], colorectal cancer [11], and etc. MiR-21 has been demonstrated as a key regulator of biological behaviors of cancer cells, including apoptosis [10], proliferation [12], EMT [13], migration and invasion [14]. Accumulating evidence demonstrates that miR-21 participated in EMT via several signal pathways. Luo et al. found that STAT3/miR-21 activation by IL-6 was associated with arsenite-induced EMT of human bronchial epithelial (HBE) cells [15]. Our previous research also suggested that inhibition of STAT3/miR21 axis with WP1066, a small-molecule STAT3 inhibitor, suppressed HNSCC cell growth and sensitized cells to cisplatin [16, 17]*.*

The neuronal kinase CDK5, which functions in migration, was recently reported to be activated in human cancers and implicated in promoting metastasis [18]. CDK5 activation regulates prostate cancer cell motility and metastatic potential [19]. Inhibition of CDK5 activity reduces the tumorigenic and metastatic properties of pancreatic cancer cells [18]. In an orthotropic xenograft model of human pancreatic cancer, inhibition of CDK5 reduces tumor growth and metastasis [18]. CDK5 functions through its regulatory subunit, p35, in multiple caner types. In medullary thyroid carcinoma (MTC), CDK5, p35 and p25 are highly expressed, and CDK5 promotes tumorigenesis and tumor progression via down-regulating its downstream target, Retinoblastoma gene (Rb) [20]. An elevated expression of CDK5 and p35 in lung cancer cells is associated with enhanced lung cancer cell migration and invasion [21].

Although miR-21 and CDK5 respectively regulates HNSCC metastasis, whether miR-21 promotes HNSCC metastasis via regulating CDK5 has not been documented in literature. In the present study, we for the first time demonstrated that miR-21 promotes HNSCC lymph node metastasis and EMT via upregulating CDK5.

## Methods

### Human tissue samples, IHC staining and ISH

A total of 60 HNSCC tumor samples were included in this study. All the patients received radical resection of tumor and neck lymph node dissection at Tianjin Medical University Cancer Institute & Hospital between January 2008 and December 2012. Ten tissues samples adjacent to tumors were also collected and used as controls. Institutional Human Subject Research Review Committee of Tianjin Medical University approved all procedures. All cases were grouped into positive or negative lymph node metastasis according to the American Joint of Cancer Committee’s Lip and Oral Cancer TNM staging system (2010 version) [22]. All tumor samples and tissues adjacent to tumor were paraffin-embedded and sectioned for routine immunohistochemistry (IHC) staining and in situ hybridization. Hybridization was performed with locked nucleic acid (LNA)-modified antisense miR-21 probe (Boster, China) and IHC for CDK5 (Cell Signaling Technology, USA), N-cadherin (Cell Signaling Technology, USA), Vimentin (BD Bio, CA, USA), β-catenin (Cell Signaling Technology, USA), STAT3/-p (Cell Signal Technology, USA), Ki-67 (Zhongshan, China), cleaved caspase 3 (Cell Signaling Technology, USA) and E-cadherin (Cell Signaling Technology, USA). The In situ hybridization (ISH) and IHC assays were performed as previously described [12].

### Cell culture and transfection

The human Hep-2 and Tca8113 cell lines were purchased from the China Center for Type Culture Collection (Wuhan, China) and maintained in modified Eagle’s medium (MEM) and RPMI-1640 (Invitrogen, USA) supplemented with 10 % fetal bovine serum (Gibco, USA). WP1066 (Calbiochem, Germany) was dissolved in DMSO (Solarbo, China) for use and storage. Hep-2 and Tca8113 cells were treated with WP1066 for 48 h at a concentration of 4.5 μM. The asON was transfected by using Lipofectamine 2000 (Invitrogen, USA) at a final concentration of 300 nmol/L and 233 nmol/L, respectively, according to the manufacturer’s instructions. 48 h after transfection, cells were harvested for total RNA or miRNAs isolation, and protein extraction. The CDK5 plasmid (CDK5(BC005115)-GV141) was synthesized by Gene Chem Company, China.

### RNA extraction and real-time PCR

Total RNAs were extracted by using the Trizol Reagent (Life technology, USA) in accordance with the manufacturer’s instructions. Both RT and PCR primers were purchased from Gene Pharma (Shanghai, China). Expression of mature miR-21 was measured using miR-21 QPCR kit (Beyotime Biotechnology, China). Relative quantification was conducted using amplification efficiencies derived from cDNA standard curves. Relative gene expressions were obtained. Quantitative analysis of change in expression levels was performed using a real-time PCR machine (7500 ABI; USA). The PCR procedure was performed on the Real-time PCR (Bio-Rad, USA) as described previously [12]. U6 was used as an internal control.

### Western blot analysis

WP1066 and asON treated Hep-2 and Tca8113 cells were collected for total protein extraction. Extracted proteins were transferred to PVDF membranes (Millipore, USA) and probed with the primary antibodies against N-cadherin (Cell Signaling Technology, USA), vimentin (BD Bio, CA, USA), β-catenin (Cell Signaling Technology, USA), p-β-catenin (Cell Signaling Technology, USA), E-cadherin (Cell Signaling Technology, USA), CDK5 (Cell Signaling Technology, USA), STAT3/-p (Cell Signal Technology, USA). β-actin (Zhongshan, China) and Histone H2A (Cell Signaling Technology, USA) was used as a loading control in total cell extracts and nuclear extracts.

### Immunofluorescence staining

For immunofluorescence staining, Hep-2 and Tca8113 cells were seeded on coverslips and fixed with 4 % paraformaldehyde (PFA, Sigma), treated with 1 % Bovine serum albumin (Equitech-Bio, USA) for 30mins and incubated with the antibodies described above overnight at 4 °C. Alexa Fluor 546Goat Anti-Rabbit IgG (1:400 dilutions, Zhongshan, China) was added for 1 h at 37 °C. DAPI reagent was used to stain the cell nuclei. The cells were visualized using FV-1000 laser scanning confocal microscopes.

### Wound healing and transwell assays

Hep-2, Tca8113 and Tca8113P160 cells layers were scratched using a 20 μl sterile pipette tip to form wound gaps. The wound location in the six-well plates was marked. Cells were photographed to record the wound width (0 h). Cells were cultured in serum-free medium. Photographs were taken after 24, 48 and 72-h at the marked wound location to measure the cell migration ability. Cell invasion assays were performed using transwell membranes coated with Matrigel (BD Bio, CA, USA). The lower chamber was filled with 10 % FBS. After 48 h, cells remaining in the upper chamber were removed with cotton swabs, while invading cells were fixed with 3 % paraformaldehyde (Santa Cruz, USA), stained with crystal violet (Solarbo, China). Cells penetrated through the polyethylene terephthalate membrane were counted in ten representative microscopic fields (200 × magnification).

### Tca8113 xenograft tumor assay

Tianjin Medical University Animal Care and Use Committee approved all animal experimental protocols. Four nude mice were injected with 10^7^ of Tca8113 cells subcutaneously. Mice were monitored daily and three out of four mice formed tumors. Ten days after xenografting, when the tumor size reached approximately 7 mm in diameter, the tumors were surgically removed, cut into pieces of 1–2 mm^3^ and re-implanted into the left inguinal region of 20 recipient mice. The mice were randomly divided into four groups (DMSO group, nsON-treated group, WP1066-treated group and asON-treated group) for perspective treatment and observation. At the end of the 23^rd^ day of observation, the mice were sacrificed and the xenograft tumors were removed for formalin fixation and preparation of paraffin-embedded sections.

### Statistical analysis

Data were analyzed using software SPSS 17.0. The interrelationship of miR-21, CDK5 and EMT related protein expression with lymph node metastasis status was analyzed using *χ*^2^ or Fisher exact test. Spearman’s correlation test was used to analyze the correlation of miR-21, CDK5 and the EMT related proteins with lymph node metastasis status. The results were expressed as mean +/- standard error (SE), which represented the average of at least three experiments and each of them was performed in triplicate. Statistics was determined using the analysis of variance test. A *p* value less than 0.05 was considered statistically significant.

### Statement of human tissue and animals experiments

We confirmed that all the protocols on human tissue examination and animal experiments were approved by the Committee of Medical Ethics at Tianjin Medical University.

## Results

### Overexpression of miR-21/CDK5 is associated with EMT and lymph node metastasis in HNSCC

**Fig. 1:**
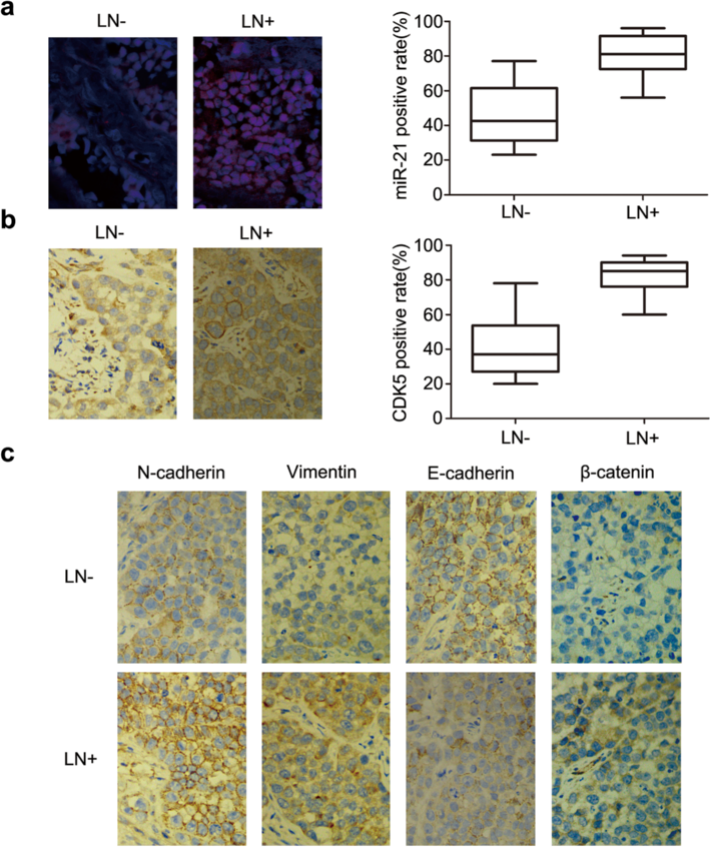
Differential expression of miR-21, CDK5 and EMT-related proteins in lymph node (LN) metastasis and LN-negative HNSCC samples. Protein expression level in lymph node with different status of metastasis is shown for **a**, MiR-21; **b**, CDK5; and **c**, N-cadherin, vimentin, E-cadherin and β-catenin

In the present study, we determined the expression level of miR-21, CDK5 and EMT-related proteins in 60 HNSCC samples. The clinical staging was determined according to American Joint of Cancer Committee (AJCC) Lip and Oral Cancer TNM staging system (2010 version). As shown in the representative IHC staining and in situ hybridization staining in Fig. 1a and b, expression of miR-21 (red) and CDK5 was elevated in lymph node positive group compared with the negative group (*χ*^2^ = 8.906, *p* =0.003; *χ*^2^ = 14.102, *p* =0.000). MiR-21 and CDK5 were significantly associated with lymph node metastatic status (R = 0.385, *p* =0.002; R = 0.527,*p* =0.000). As shown in Fig. 1c and Additional file 1: Figure S2B, the expressions of STAT3 and EMT markers were enhanced in lymph node positive group with significant correlation to lymph node metastatic status in HNSCC samples, for N-cadherin (*X*^2^ = 21.365,*p* =0.000;R = 0.647, *p* =0.000), vimentin (*X*^2^ = 7.358, *p* =0.007;R = 0.350, *p* =0.006), E-cadherin (*X*^2^ = 21.609, *p* =0.000;R = -0.645, *p* =0.000), β-catenin (*X*^2^ = 5.742, *p* =0.017;R = 0.354, *p* =0.002). All HNSCC samples were analyzed using Kaplan-Meier analysis and a multivariate COX regression model. Analysis showed that the expression of miR-21, CDK5, EMT markers, and lymph node metastasis are closely correlated (Additional file 2: Table S1). Univariate analysis showed significant relationships between the Overall survival (OS) and higher T stage, high expression of CDK5, MiR-21, N-cadherin, Vimentin, low expression of E-cadherin, and lymph node metastasis (*p* < 0.05). Furthermore, T stage (HR: 1.248-3.392; *p* < 0.05), MiR-21expression (HR: 1.448-13.607; *p* < 0.05), and N-cadherin expression (HR: 1.795-38.098; *p* < 0.05) were identified as the independent factors of the OS, which were analyzed by Multivariate Analysis. Protein level of CDK5 and EMT markers were verified by Western blot in the aforementioned corresponding samples of HNSCC (Additional file 3: Figure S1B).

### WP1066 inhibited MiR-21/CDK5 in HNSCC cell lines Hep-2 and Tca8113 in vitro

**Fig. 2:**
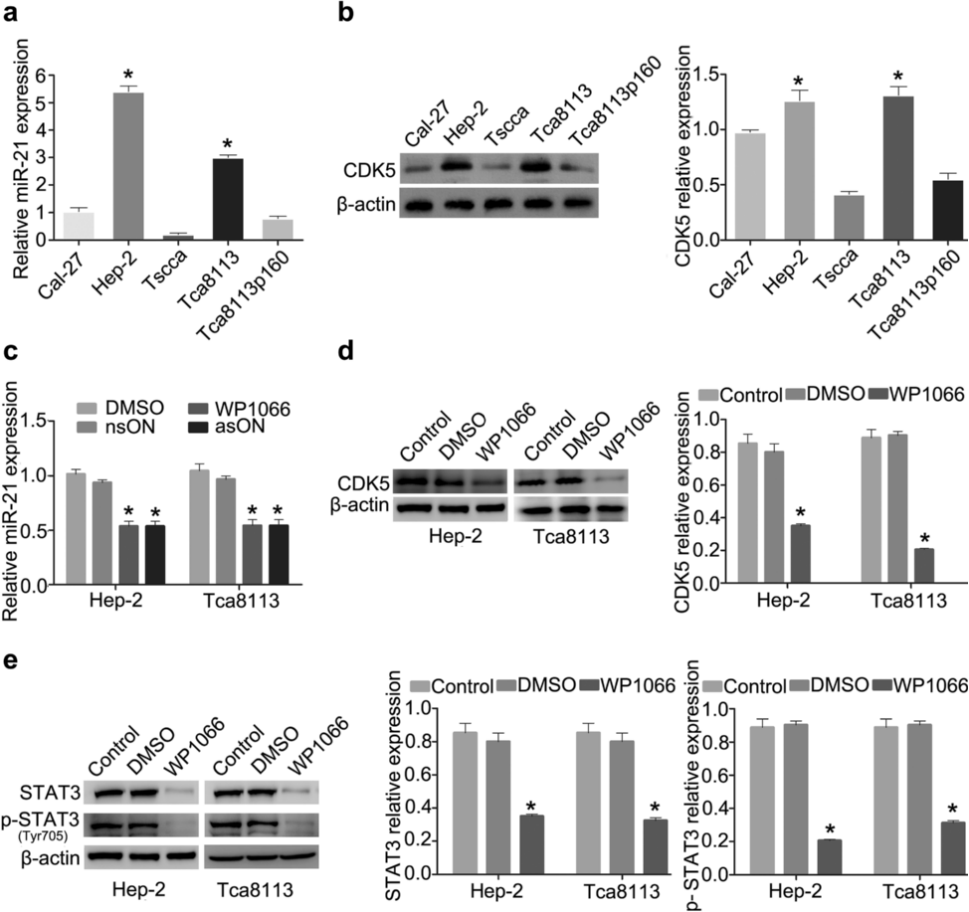
WP1066 inhibits MiR-21/CDK5 in HNSCC cell lines Hep-2 and Tca8113. **a** MiR-21 expression in Hep-2 and Tca8113 cells. **b** CDK5 expression in Hep-2 and Tca8113 cells. **c** MiR-21 expression level alteration after WP1066 or asON treatment. **d** CDK5 expression in Hep-2 and Tca8113 cells treated with WP1066. **e** STAT3 and pSTAT3 expression after WP1066 treatment in Hep-2 and Tca8113 cells

To investigate the involvement of miR-21 and CDK5 in HNSCC, we examined the expression of miR-21 in different HNSCCs cell lines (Cal-27, Hep-2, Tscca, Tca8113, and Tca8113p160). By qRT-PCR and Western blot analysis, we showed that the expression levels of miR-21 and CDK5 were remarkably higher in Hep-2 and Tca8113 cells than other HNSCCs cell lines, as showed in Fig. 2a and b. We also validated the protein level of STAT3 and pSTAT3 in all the tested cell lines, showing significantly increased pSTAT3 in Hep-2 and Tca8113 cells (Additional file 3: Figure S1A), suggesting a positive correlation of expression of miR-21, CDK5, STAT3 and activated pSTAT3. Therefore, Hep-2 and Tca8113 cells were chosen for further investigation.

To knockdown miR-21 in Hep-2 and Tca8113 cells, we used WP1066, a specific STAT3 small-molecule inhibitor, and antisense oligonucleotide (asON) to suppress miR-21 activity. MiR-21 relative expression was examined after asON exposure for optimal time and dose, the nsON was used as a control group. The optimal time and dose of WP1066 treatment in Hep-2 and Tca8113 cells were demonstrated in our previous report [16, 17]. As shown in Fig. 2c, miR-21 expression was significantly decreased after WP1066 or asON treatment. Meanwhile, CDK5 expression was suppressed by WP1066 in both Hep-2 and Tca8113 cells (Fig. 2d). Consistent with our previous report, STAT3 and pSTAT3 expression were effectively inhibited by WP1066 when compared with parental and DMSO-treated Hep-2 and Tca8113 cells (Fig. 2e).

### Reduction of miR-21 impaired HNSCC migration, invasion and EMT by regulating CDK5/p35 in vitro

**Fig. 3:**
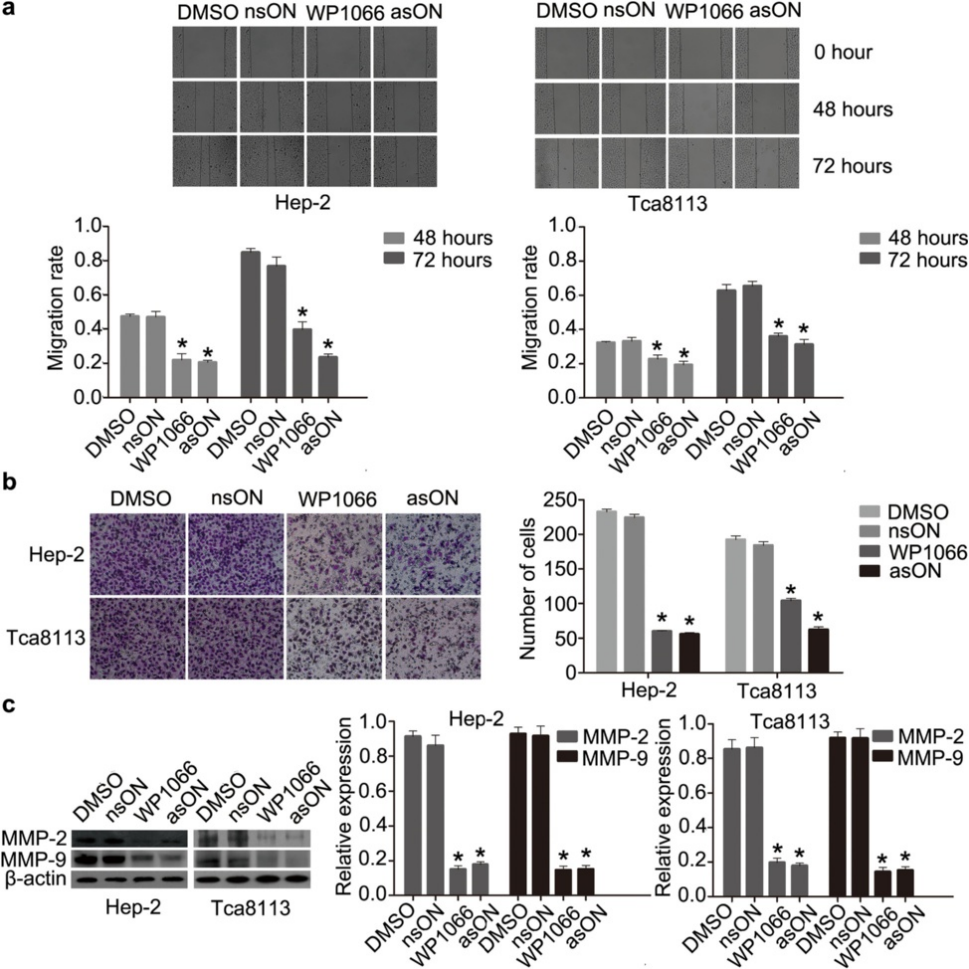
miR-21 blockade reduces migration and invasion of Hep-2 and Tca8113 cells in vitro. **a** Wound-healing assay of Hep-2 and Tca8113 cells. **b** Transwell invasion of Hep-2 and Tca8113 cells. Cells were placed in the upper well of the transwell plate and FBS was added into the lower well of the serum-free media. After 48 h of incubation, the percentage of invaded cells was calculated. (original magnification, x200). **c** Western blotting of MMP-2/-9 expression in Hep-2 and Tca8113 cells after WP1066 and asON treatment

**Fig. 4:**
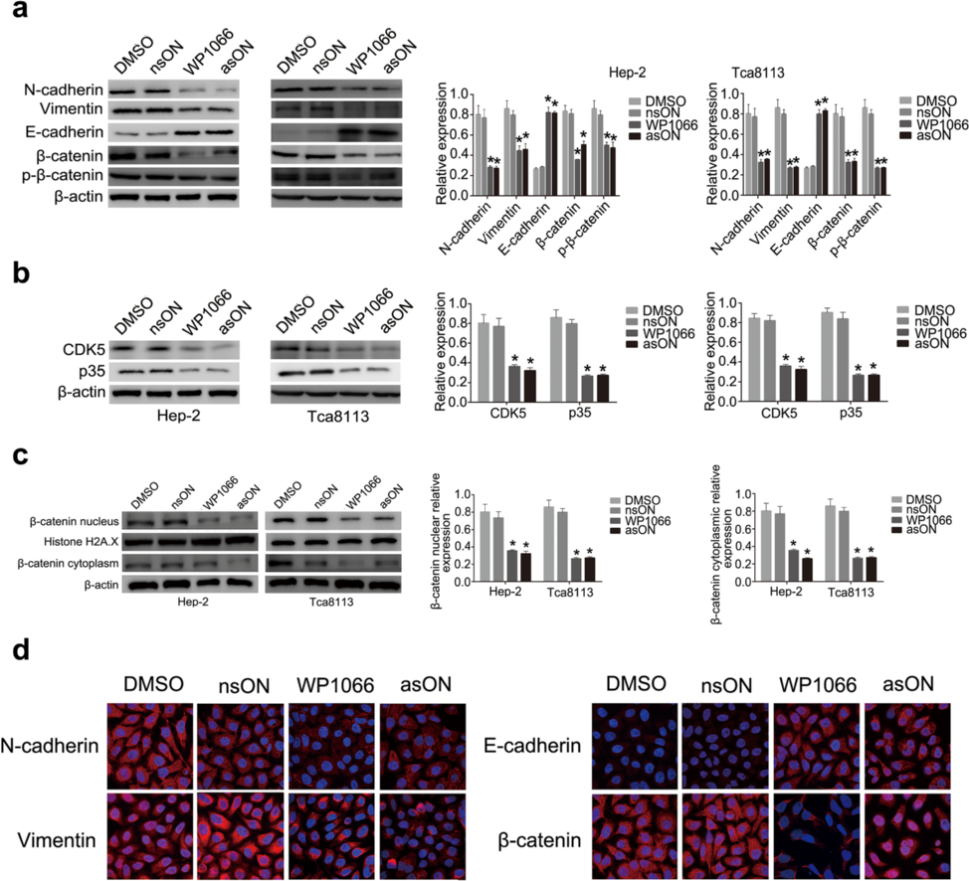
miR-21 blockade reverses EMT by regulating CDK5/p35 in HNSCC cells in vitro. **a** Expression of EMT-related proteins after asON or WP1066 treatment, analyzed by western blot with normalization to β-actin. **b** Expressions of CDK5 and p35 after asON and WP1066 treatment, determined by western blot with normalization to β-actin. **c** β-catenin level in nucleus and cytoplasm, analyzed by western blot with normalization to Histone H2A. **d** Subcellular location of EMT related markers after asON or WP1066 treatment, observed by immunofluorescence staining (original magnification, x100)

We used asON and WP1066 to antagonize miR-21 in Hep-2 and Tca8113 cells. nsON and DMSO were used as controls. After 48 h of treatment, scratch and transwell chamber assay were employed to measure antagonistic effect in in vitro cell migration and invasion. The number of migrating and invading cells in asON and WP1066-treated cultures were reduced relative to the control cells (*p* < 0.05, Fig. 3a, b), demonstrating that both asON and WP1066 could impair the ability of Hep-2 and Tca8113 cells migration and invasion. Moreover, we examined the MMP-2/MMP-9 expression levels in Hep-2 and Tca8113 cells after WP1066 and asON exposure by Western blot. As shown in Fig. 3c, both MMP-2 and MMP-9 expression was decreased in WP1066 and asON groups. All these results demonstrated that knockdown of miR-21 had a suppressive effect on the migration and invasion ability of HNSCC cells in vitro. We then focused on the effect of knocking-down miR-21 on the EMT-phenotypes of Hep-2 and Tca8113 cells. Western blot showed that in both Hep-2 and Tca8113 cells treated with asON and WP1066, E-cadherin expression level was elevated while the N-cadherin expression was attenuated. In addition, a decreased expression of vimentin was also observed. Importantly, the β-catenin expression in nucleus and cytoplasm were both decreased (Fig. 4a, c), suggesting that antagonism of miR-21 by asON and WP1066 led to EMT reversal in Hep-2 and Tca8113 cells. Immunofluorescence (IF) staining showed a notable reduction of nuclear and cytoplasmic β-catenin in asON and WP1066 groups compared with the control group. We also observed inhibition of N-cadherin and vimentin in asON and WP1066-treated cells, compared with untreated cells. Meanwhile, E-cadherin exhibited opposite expression after treatment (Fig. 4d, Additional file 1: Figure S2A). The alteration of these EMT-related proteins after asON or WP1066 exposure suggests that the EMT in Hep-2 and Tscca8113 cells was suppressed.

To further elucidate that inhibition of miR-21 reversed EMT through CDK5/p35, CDK5 and p35 expression were detected by Western blot analysis. In Fig. 4b, it showed that CDK5 and p35 were significantly down regulated by asON and WP1066 in Hep-2 and Tca8113 cells, suggesting miR-21 regulates EMT process in a CDK5/p35 dependent manner.

### Reduction of miR-21 results in reversal of EMT by regulating CDK5 in HNSCC in vitro and in vivo

**Fig. 5:**
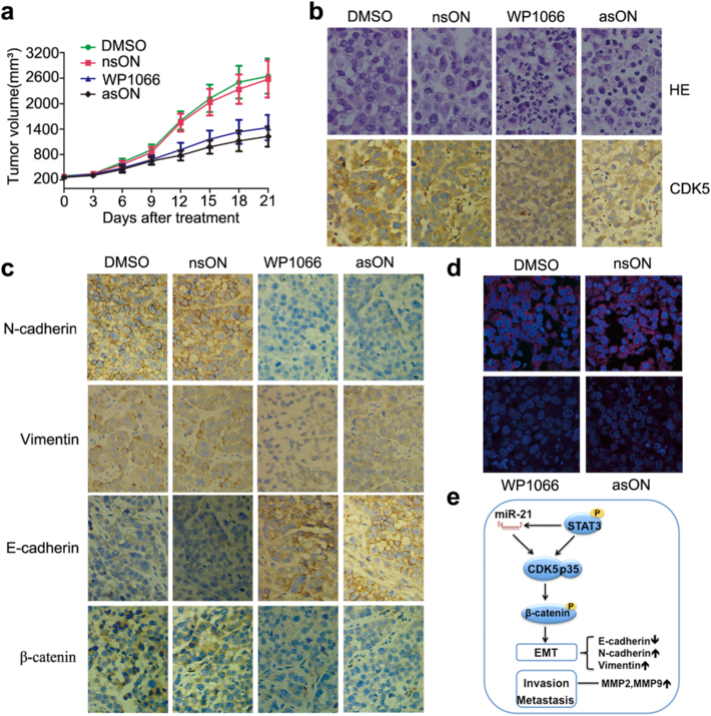
miR-21 blockade leads to reversal of EMT via regulating CDK5 in vivo*.* **a** The growth curves of Tca8113 xenograft tumors treated with DMSO, nsON, WP1066, and asON. **b** HE and IHC staining of CDK5. **c** IHC staining for alteration of EMT markers (original magnification, x400). **d** ISH for miR-21 inhibition by WP1066 and asON. **e** A hypothesis schema demonstrating the relationship among miR-21, STAT3 and CDK5/p35 in EMT of HNSCC

In vitro experiments demonstrated that miR-21 reversed EMT process via CDK5/p35. We thus employed a Tca8113 xenograft tumor model to confirm this effect in vivo. The growth rates in WP1066 and asON group were suppressed compared to the control and DMSO groups (Fig. 5a). The impaired tumor growth is a consequence of both decreased proliferation and increased apoptosis as demonstrated by IHC staining of the tumors for Ki-67 and cleaved caspase 3, respectively (Additional file 1: Figure S2C). ISH analysis showed that miR-21 was inhibited by WP1066 and asON (Fig. 5d). IHC staining and western blot demonstrated decreased expression levels of STAT3, pSTAT3, CDK5, N-cadherin, vimentin and β-catenin and increased expression of E-cadherin, as observed in both WP1066- and asON-treated groups in xenograft tumors (Fig. 5b, c, Additional file 1: Figure S2C, Additional file 3: Figure S1D). We further validated that CDK5 is a required component for miR-21 to regulate EMT and invasion. CDK5 was overexpressed in Tca8113p160 cells, and we observed increased expression of EMT markers (Additional file 3: Figure S1E), enhanced migration and invasion in Tca8113p160 cells (Additional file 3: Figure S1 F, G). Moreover, CDK5 overexpression reverted the inhibition of EMT markers expression by asON and WP1066 in Hep-2 and Tca8113 cells (Additional file 3: Figure S1C), suggesting that CDK5 overexpression in HNSCC cells is sufficient to induce tumor cell motility via modulating EMT. These results not only support co-regulation of miR-21 and CDK5, but also establish a causal link connecting miR-21, CDK5 and cancer cell migration, invasion and metastasis.

## Discussion

In the present study, miR-21 inhibition was shown to reverse the EMT process in HNSCC cells by regulating CDK5/CDK5R1 (p35) with antisense oligonucleotide (asON) or a STAT3 inhibitor WP1066.

The tumor cells undergoing EMT is a characteristic of acquiring migratory and invasive mesenchymal phenotypes. Diverse upstream signaling is implicated in this process. Of particular importance, the Wnt pathway plays an essential role by triggering the translocation of β-catenin to the nucleus, leading to the activation of EMT-related target genes and ultimately promoting EMT [23]. Zhang et al. also reported that activation of Wnt/β-catenin pathway is potentially responsible for the EMT phenotype in SGC7901/ADR cells [24]. In our study, the EMT reversal is supported by notable alteration of nucleus-cytoplasm expression of β-catenin by IF and IHC.

MicroRNAs are defined as a subset of small non-coding RNA, which are less than 22 nucleotides long. In general, they function as negative regulators of target genes at transcriptional and post-transcriptional level. Some of them act as tumor-suppressors while other may act as tumor-promoters. Recently, several miRNAs such as miR-21, have been shown to be involved in EMT [25, 26]. Our previous study has demonstrated that reduction of miR-21 induces glioma cell apoptosis via activating caspases 9 and 3 [10]. Downregulated miR-21 inhibits the growth of human glioblastoma cells through EGFR pathway [12]. However, the mechanisms by which miR-21 regulates EMT is not clear. Han et al. showed that AKT and ERK1/2 pathways are required for miR-21 in mediating EMT and CSC phenotype by targeting PTEN [27]. Similarly, miR-21 inhibits its targets-PTEN and sulfatase-1 (hSulf-1) expression in hepatocellular carcinoma (HCC) cells and finally enhances HCC cell proliferation and movement [28]. Meanwhile, miR-21 directs EMT and enhances the invasive potential of transformed human bronchial epithelial cells by targeting PDCD4 [29]. However, Wang et al. found that ectopic expression of miR-21 could overcome TGF-β growth-inhibitory effect but had no effect on EMT of TGF-β in HaCaT cells [13]. Our results suggest that knockdown of miR-21 could reverse the process of EMT in HNSCC cells via CDK5/p35.

CDK5 is a small serine/threonine cyclin-dependent kinase that belongs to the family of CDKs. In contrast to the cell cycle-related CDKs (e.g. CDKs 1, 2, 4, or 6), CDK5 is not implicated in cell cycle control [30]. Instead, CDK5 is involved in neuronal migration and development during embryogenesis [31]. In neurons, CDK5 has been reported to interact with β-catenin through p35, and regulates the affinity of β-catenin for cadherin by altering the phosphorylation levels of β-catenin [32, 33]. Demelash et al. reported that induction of CDK5 regulated lung cancer cell migration through Achaete-scute complex homologue-1 (ASH1). ASH1 directly modulates the expression ofp35 gene in small cell lung cancer cells [21]. Furthermore, CDK5 can also regulate endothelial cell migration and angiogenesis, suggesting CDK5 may serve as a novel target for anti-angiogenic therapy [34].

It was reported that miR-21 targets a number of important molecules such as PTEN, PDCD4, TIMP3 and TMP1 [35, 36]. CDK5 is also involved in neuregulin-dependent activation of phosphatidylinositol 3-kinase (PI3K) and Akt activity [37], and promotes pancreatic β-cell survival via Fak-Akt signaling pathways [38]. In the present study, we attempted to demonstrate that CDK5 was the functional target of miR-21. Our results suggest that the miR-21/CDK5 axis could partially mediate tumor cell mobility and EMT by targeting STAT3 in HNSCC. Moreover, our previous study revealed that abnormal activation of miR-21/CDK5 axis was associated with breast cancer lymph node metastasis [39].

Although several studies have established that miR-21 could promote EMT in tumor cells by a number of mechanisms, it is for the first time that we demonstrate CDK5 regulation by miR-21 to promote metastasis in HNSCC. Our findings demonstrated the importance of miR-21/CDK5signaling in tumor cell migration and invasion in vitro and in vivo. Our studies suggest the intrinsic connection between CDK5 and miR-21 in modulating EMT in HNSCC. Based on these observations, we believe that targeting miR-21/CDK5 might be a novel and feasible approach for managing metastasis from HNSCC. Innovative strategies targeting miR-21 and CDK5 are currently being pursued in our labs.

## Conclusions

MiRNA-21 promotes metastasis of HNSCC via regulating CDK5/CDK5R1 (p35) to enhance EMT. Blocking miR-21 with STAT3 inhibitor WP1066 effectively reversed EMT.

## Additional files

Additional file 1: Figure S2. A, Immunofluorescence showing subcellular location of E-cadherin, N cadherin and beta-catenin after asON or WP1066 treatment. B, IHC staining of STAT3 and vimentin in tumor tissue sections in positively correlation to lymph node metastasis of HNSCC patients. C, IHC showing the altered expression of STAT3/pSTAT3, Ki67 and cleaved caspase 3 after DMSO, nsON, WP1066, and asON treatments in Tca8113 xenograft tumors. (JPG 3251 kb) Additional file 2: Table S1. The K-M analysis and COX regression analysis of 60 HNSCC patients. (DOCX 21 kb) Additional file 3: Figure S1. A, The expression of STAT3 and pSTAT3 protein in five HNSCC cell lines examined by Western blot. B, Expression of CDK5 and EMT markers in relation to the status of lymph node metastasis of HNSCC tissues. C, Ectopic overexpression of CDK5 in Hep-2 and Tca8113 cells and resulting revert of EMT after miR-21 silencing or WP1066 treatment. D, Expression of CDK5 and EMT markers in mouse xenografts tissues after WP1066 or asON treatment. E, Expressions of EMT markers after CDK5 plasmid transfection in Tca8113P160 cells. F, Induction of tumor cell motility and EMT after ectopic CDK5 overexpression in transwell assay in Tca8113p160 cells. G, Induction of tumor cell motility and EMT after ectopic CDK5 overexpression in scratch assay in Tca8113p160 cells. (JPG 16931 kb)

## Abbreviations

EMT: Epithelial-Mesechymal Transition
miR-21: microRNA-21
HNSCC: Head and neck squamous cell carcinoma
CDK5: Cyclin-dependent kinase 5
STAT3: Signal transducers and activators of transcription 3
asON: Antisense oligonucleotide
nsON: nonsense oligonucleotide
DMSO: dimethyl sulfoxide
HBE: Human bronchial epithelial
MTC: Medullary thyroid carcinoma
CSC: Cancer stem cell
Rb: Retinoblastoma
BSA: Bovine serum albumin
IHC: Immunohistochemistry
FISH: Fluorescence in situ hybridization
RT-PCR: Reverse transcription polymerase chain Reation
PVDF: Polyvinylidene Fluoride
PDCD4: Programmed cell death 4
SCLCs: Small cell lung cancers
