# Supplementary figures and images for: Targeting STAT3/miR-21 axis inhibits epithelial-mesenchymal transition via regulating CDK5 in head and neck squamous cell carcinoma
Source: Mol Cancer. 2015 Dec 21;14:213. doi: 10.1186/s12943-015-0487-x (PMC4687320; doi:10.1186/s12943-015-0487-x)

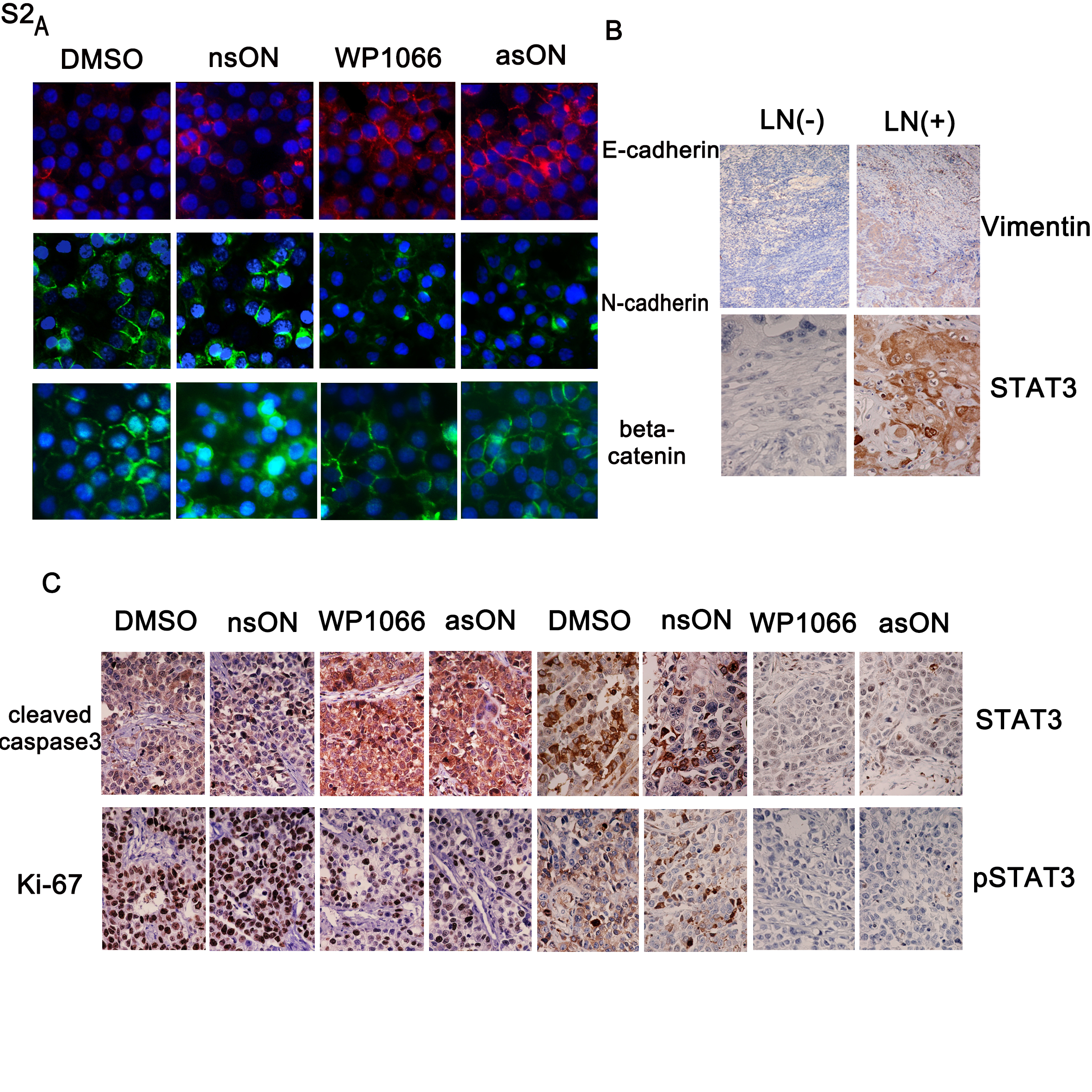

Supplement: Additional file 1: Figure S2. — A, Immunofluorescence showing subcellular location of E-cadherin, N cadherin and beta-catenin after asON or WP1066 treatment. B, IHC staining of STAT3 and vimentin in tumor tissue sections in positively correlation to lymph node metastasis of HNSCC patients. C, IHC showing the altered expression of STAT3/pSTAT3, Ki67 and cleaved caspase 3 after DMSO, nsON, WP1066, and asON treatments in Tca8113 xenograft tumors. (JPG 3251 kb) [file 12943_2015_487_MOESM1_ESM.jpg]
