# Supplementary material for: Targeting STAT3/miR-21 axis inhibits epithelial-mesenchymal transition via regulating CDK5 in head and neck squamous cell carcinoma
Source: Mol Cancer. 2015 Dec 21;14:213. doi: 10.1186/s12943-015-0487-x (PMC4687320; doi:10.1186/s12943-015-0487-x)
Supplement: Additional file 2: Table S1. — The K-M analysis and COX regression analysis of 60 HNSCC patients. (DOCX 21 kb) [file 12943_2015_487_MOESM2_ESM.docx]

**Supplementary Table.1 The K-M analysis and COX regression analysis of 60 HNSCC patients.**

| Variables | N | Univariate Analysis | | Multivariate Analysis | | | |
| --- | --- | --- | --- | --- | --- | --- | --- |
|  |  | **Chi-square** | **P Value** | **Hazard Ratio** | **P Value** | **95%CI** | |
| Gender  Male  Female | 55  5 | 2.419 | 0.120 |  |  | | |
| Age  <70  ≥70 | 52  8 | 0.396 | 0.529 |  |  | | |
| Smoking  Yes  No | 49  11 | 0.916 | 0.339 |  |  | | |
| Alcohol  Yes  No | 27  33 | 1.803 | 0.179 |  |  | | |
| Tumor Localization  Larynx  Oropharynx  Oral Cavity | 24  9  27 | 1.075 | 0.584 |  |  | | |
| T stage  T1  T2  T3  T4 | 27  12  12  9 | 11.306 | 0.010 | 2.057 | 0.005 | | 1.248-3.392 |
| CDK5  High  Low | 24  36 | 4.593 | 0.032 | 1.706 | 0.412 | | 0.476-6.114 |
| MiR-21  High  Low | 27  33 | 6.609 | 0.010 | 4.439 | 0.009 | | 1.448-13.607 |
| E-cadherin  High  Low | 41  19 | 4.314 | 0.038 | 0.592 | 0.340 | | 0.202-1.736 |
| N-cadherin  High  Low | 47  13 | 9.755 | 0.002 | 8.270 | 0.007 | | 1.795-38.098 |
| Vimentin  High  Low | 28  32 | 4.573 | 0.032 | 1.607 | 0.429 | | 0.496-5.200 |
| Beta-catenin  High  Low | 19  41 | 2.719 | 0.099 |  |  | | |
| Lymph node metastasis  Yes  No | 12  48 | 4.114 | 0.043 | 0.255 | 0.141 | | 0.041-1.570 |
